# Patients' preferences for adjuvant chemotherapy in early-stage breast cancer: is treatment worthwhile?

**DOI:** 10.1054/bjoc.2001.1836

**Published:** 2001-06

**Authors:** S J T Jansen, J Kievit, M A Nooij, J C J M de Haes, I M E Overpelt, H van Slooten, E Maartense, A M Stiggelbout

**Affiliations:** Department of Medical Decision Making, K6-R, Leiden University Medical Center, PO Box 9600, 2300 RC Leiden, The Netherlands; Department of Clinical Oncology, K1-P, Leiden University Medical Center, PO Box 9600, 2300 RC Leiden, The Netherlands; Department of Medical Psychology, Academic Medical Center, PO Box 22660, 1100 DD Amsterdam, The Netherlands; Department of Internal Medicine, Diaconessen Hospital, PO Box 9650, 2300 RD Leiden, The Netherlands; Department of Internal Medicine, Reinier de Graaf Gasthuis, PO Box 5012, 2600 GA Delft, The Netherlands

**Keywords:** preferences, breast cancer, chemotherapy, cognitive dissonance reduction, shared decision-making

## Abstract

When making decisions about adjuvant chemotherapy for early-stage breast cancer, costs and benefits of treatment should be carefully weighed. In this process, patients' preferences are of major importance. The objectives of the present study were: (1) to determine the minimum benefits that patients need to find chemotherapy acceptable, and (2) to explore potential preference determinants, namely: positive experience of the treatment, reconciliation with the treatment decision, and demographic variables. Preferences were elicited from patients scheduled for adjuvant chemotherapy (chemotherapy group: *n* = 38) before (T _1_), during (T _2_), and 1 month after chemotherapy (T _3_), and were compared to responses from patients not scheduled for chemotherapy (no-chemotherapy group: *n* = 38). The patients were asked, for a hypothetical situation, to indicate the minimum benefit (in terms of improved 5-year disease-free survival) to find adjuvant chemotherapy acceptable. In the chemotherapy group, the median benefit was 1% at all 3 measurement points. In the no-chemotherapy group the attitude towards chemotherapy became more negative over time, although not statistically significantly so (T _1_: 12%, T _2_: 15%, T _3_: 15%;*P* = 0.10). At all measurement points, the patients in the chemotherapy group indicated that they would accept chemotherapy for significantly (*P*< 0.01) less benefit than the patients in the no-chemotherapy group. Of the demographic variables, age was related to preferences, but only at T _2_ and only in the no-chemotherapy group. The more positive attitude towards chemotherapy and the stability of preferences in the chemotherapy group indicated that reconciliation with the treatment decision was a more important determinant of patients' preferences than positive experience of the treatment. © 2001 Cancer Research Campaign http://www.bjcancer.com


*British Journal of Cancer* (2001) **84**(12), 1577–1585
© 2001 Cancer Research Campaign
doi: 10.1054/ bjoc.2001.1836, available online at http://www.idealibrary.com on IDEAL®

http://www.bjcancer.com

### Patients' preferences for adjuvant chemotherapy in early-stage breast cancer: is treatment worthwhile?

**SJT Jansen[1,2], J Kievit[1], MA Nooij[2], JCJM de Haes[3], IME Overpelt[1,2], H van Slooten[4], E Maartense[5] and AM Stiggelbout[1]**

[1]Department of Medical Decision Making, K6-R, Leiden University Medical Center, PO Box 9600, 2300 RC Leiden, The Netherlands; [2]Department of Clinical Oncology, K1-P, Leiden University Medical Center, PO Box 9600, 2300 RC Leiden, The Netherlands; [3]Department of Medical Psychology, Academic Medical Center, PO Box 22660, 1100 DD Amsterdam, The Netherlands; [4]Department of Internal Medicine, Diaconessen Hospital, PO Box 9650, 2300 RD Leiden, The Netherlands; [5]Department of Internal Medicine, Reinier de Graaf Gasthuis, PO Box 5012, 2600 GA Delft, The Netherlands

**Summary** When making decisions about adjuvant chemotherapy for early-stage breast cancer, costs and benefits of treatment should be carefully weighed. In this process, patients' preferences are of major importance. The objectives of the present study were: (1) to determine the minimum benefits that patients need to find chemotherapy acceptable, and (2) to explore potential preference determinants, namely: positive experience of the treatment, reconciliation with the treatment decision, and demographic variables. Preferences were elicited from patients scheduled for adjuvant chemotherapy (chemotherapy group: $n = 38$) before ($T_1$), during ($T_2$), and 1 month after chemotherapy ($T_3$), and were compared to responses from patients not scheduled for chemotherapy (no-chemotherapy group: $n = 38$). The patients were asked, for a hypothetical situation, to indicate the minimum benefit (in terms of improved 5-year disease-free survival) to find adjuvant chemotherapy acceptable. In the chemotherapy group, the median benefit was 1% at all 3 measurement points. In the no-chemotherapy group the attitude towards chemotherapy became more negative over time, although not statistically significantly so ($T_1$: 12%, $T_2$: 15%, $T_3$: 15%; $P = 0.10$). At all measurement points, the patients in the chemotherapy group indicated that they would accept chemotherapy for significantly ($P < 0.01$) less benefit than the patients in the no-chemotherapy group. Of the demographic variables, age was related to preferences, but only at $T_2$ and only in the no-chemotherapy group. The more positive attitude towards chemotherapy and the stability of preferences in the chemotherapy group indicated that reconciliation with the treatment decision was a more important determinant of patients' preferences than positive experience of the treatment. © 2001 Cancer Research Campaign http://www.bjcancer.com

**Keywords**: preferences; breast cancer; chemotherapy; cognitive dissonance reduction; shared decision-making

In women with early-stage breast cancer, adjuvant chemotherapy may be given in addition to breast surgery and post-operative radiotherapy. A recent overview of randomized trials (EBCTCG, 1998) showed that in women under the age of 50 with axillary-node-positive breast cancer, the use of adjuvant chemotherapy increases the mean estimated 5-year disease-free survival by 15.2% (from 41.9% to 57.1%). However, chemotherapy may be associated with side effects such as nausea, hair loss and fatigue. Therefore, the choice for adjuvant chemotherapy involves a trade-off between the reduction in the risk of recurrence and the potential deterioration in quality of life during and shortly after treatment (Dodwell, 1998).

The question is whether the application of adjuvant chemotherapy in the treatment of early-stage breast cancer is worthwhile (Dodwell, 1998). Nowadays, the use of adjuvant chemotherapy for node-positive breast cancer in premenopausal women is generally considered to be worthwhile because of the clinical benefits (Fisher et al, 1997). The important issue is 'how low to go' in applying adjuvant chemotherapy (Pritchard, 1997). For example, women with node-negative disease between 50 and 69 years of age have a lower risk of recurrence compared to younger women with node-positive disease and subsequently their benefit from chemotherapy is smaller in absolute terms, namely an increase in the mean estimated 5-year disease-free survival of 6.3% (from 70.3% to 76.6%; EBCTCG, 1998).

Studies have shown variations in the prescription of adjuvant chemotherapy, e.g., between groups of American and European oncologists and among different oncologists within those groups (Rajagopal et al, 1994), among physicians in the UK (Sainsbury et al, 1995) and in the Netherlands (Stiggelbout et al, 2000), among different groups of doctors (general practitioners, radio-therapists, oncologists) and among different specialists within those groups (Slevin et al, 1990). These studies show that the trade-off between clinical benefits and the side effects of adjuvant chemotherapy entails a difficult and complex decision process. In this process, the patients' own preferences and opinions are highly relevant. The emphasis on patient autonomy and increasing shared decision-making requires that the preferences and opinions of patients regarding their treatment are assessed more explicitly.

Patients' preferences for treatments can be elicited by means of a so-called treatment preference method or probability trade-off method. This method requires the respondent to consider the side effects of various treatment options together with the possible outcomes of those treatments and the probabilities of obtaining those outcomes (Llewellyn-Thomas et al, 1996). For a more elaborate discussion of the application of this approach in clinical

*Received 5 September 2000*
*Received 19 February 2001*
*Accepted 19 March 2001*

*Correspondence to:* SJT Jansen decision-making, see Llewellyn-Thomas et al (1996) and Llewellyn-Thomas (1997).

The literature has shown that there are several determinants that may affect patients' treatment preferences. Previous studies have shown that patients are more willing than medical staff or the general public to accept aggressive treatment in exchange for small benefits (O'Connor, 1989; Slevin et al, 1990; Brundage et al, 1997). Thus, those who are actually ill are more likely to consider treatment acceptable. Other studies have shown that respondents tend to favour treatments that they had already experienced (Yellen et al, 1994; McQuellon et al, 1995; Cullen et al, 1996; Stiggelbout et al, 1996; Lindley et al, 1998; Nieuwkerk et al, 1998). 2 explanations have been proposed for this observation. First, the patients' satisfaction with the treatment, thus positive experience of the treatment (Yellen et al, 1994; Cullen et al, 1996; Stiggelbout et al, 1996; Lindley et al, 1998). Lindley et al (1998) explain that the patients who had experienced chemotherapy in the past might be less fearful of its potential negative effects and more comfortable with their ability to cope with the physical, social and emotional aspects of treatment. This explanation will be referred to as 'positive experience of the treatment'.

The second explanation is based on a cognitive mechanism of adaptation that people are known to use to justify the way in which they were managed, also known as cognitive dissonance reduction (Cullen et al, 1996; Lindley et al, 1998; Nieuwkerk et al, 1998). Patients may have a desire to believe that the prior decision (for treatment or no treatment) was the correct one (Lindley et al, 1998). This may make patients reluctant to reflect upon an alternative that was not proposed or that was turned down, but might have been preferable. This explanation will be referred to as 'reconciliation with the treatment decision'.

Other studies have shown that treatment preferences may be affected by demographic variables: younger age (Yellen et al, 1994; McQuellon et al, 1995; Brundage et al, 1997), living with others (e.g., a husband or a child; Yellen and Cella, 1995), being a parent (Cullen et al, 1996), and having children living at home (Yellen and Cella, 1995). All these variables are related to more willingness to accept treatment.

In our study, first, we wanted to determine the minimum benefit, in terms of improved 5-year disease-free survival, that early-stage breast cancer patients need before they are willing to accept adjuvant chemotherapy, and see how this compares to the benefit reported in the literature. For this purpose, we elicited preferences for chemotherapy in early-stage breast cancer patients.

Secondly, we wanted to study the impact of potential determinants for the preferences: (1) positive experience of the treatment, (2) reconciliation with the treatment decision, and (3) demographic variables. Specifically, to distinguish the first 2 determinants, we elicited treatment preferences longitudinally from patients scheduled for chemotherapy (chemotherapy group) before, during, and after chemotherapy, and from patients not scheduled for chemotherapy (no-chemotherapy group) at the same points in time.

Our hypothesis was that if having had positive experience with chemotherapy determines preferences, then patients from both groups should respond in the same way before the start of chemotherapy as none have any experience with chemotherapy. Subsequently, the preferences of treated patients should become more positive during chemotherapy while the responses of the no-chemotherapy patients should remain the same. If reconciliation with the treatment decision determines the preferences, then we would expect that the preferences of the patients in the chemotherapy group would be more positive towards chemotherapy before the actual start of the treatment than the preferences of patients not scheduled for chemotherapy. Finally, we examined whether the preferences were affected by demographic variables mentioned in the literature: age, living with others, being a parent and having children living at home.

### PATIENTS AND METHODS

### Patients

Between June 1996 and November 1999, consecutive early-stage breast cancer patients were recruited from 5 hospitals in the west of The Netherlands. Exclusion criteria were: the inability to write or speak Dutch, previous personal experience with chemotherapy, or a history of psychiatric illness. In addition, women under the age of 56 years with 4 or more positive lymph nodes were excluded from the study because they were eligible to participate in a trial involving autologous bone marrow transplantation.

All patients were treated according to treatment protocol and as a result the 2 groups (chemotherapy and no-chemotherapy) will differ in age and premenopausal status: The chemotherapy group consisted of pre- or perimenopausal early-stage breast cancer patients (node-positive and high-risk node-negative) who had already consented to undergo chemotherapy. This was because we did not want to interfere with personal treatment decisions. The no-chemotherapy group included postmenopausal women with positive or negative lymph nodes and pre- or perimenopausal women with low-risk node-negative disease, who had not been advised to have adjuvant chemotherapy. Some were scheduled for hormonal therapy (Tamoxifen).

Patients in the chemotherapy group were matched with patients in the no-chemotherapy group on the basis of: personal experience with radiotherapy, type of prior breast surgery (lumpectomy versus mastectomy), time between surgery and first interview, interviewer (1 of 3), and hospital in which treatment took place. There were no obvious reasons to believe that these 5 characteristics would influence the patients' preferences towards adjuvant chemotherapy. However, we attempted to make the 2 groups as similar as possible, except for the chemotherapy–no-chemotherapy distinction, in order to make this latter comparison as unconfounded as possible. We assumed that experiencing hormonal therapy within the no-chemotherapy group would not influence the patients' preferences for adjuvant chemotherapy.

The patients were interviewed 3 times: shortly before ($T_1$), during ($T_2$), and one month after completion of chemotherapy ($T_3$). The second interview was held in the middle of treatment, in the week before the third course for patients treated with 4 courses and in the week before the fourth course for patients treated with 6 courses of chemotherapy. The patients in the no-chemotherapy group were interviewed at comparable intervals. The same interviewer performed all 3 interviews with each patient.

### Treatment preference instrument

The treatment preference method used was an adapted version of the decision board described by Levine et al (1992). Firstly, patients were presented with hypothetical scenarios of the 2 treatment choices: 'no adjuvant chemotherapy, regular check ups' or 'adjuvant chemotherapy, regular check ups' (see the Appendix).

 © 2001 Cancer Research Campaign

The adjuvant chemotherapy scenario was based on the literature, the expertise of 6 clinical oncologists, and the experiences of 5 breast cancer patients who were undergoing or who had recently undergone adjuvant chemotherapy. The development of the scenario is reported elsewhere (Jansen et al, 2000). The scenario described the treatment programme as well as the potential side effects.

Second, the possible outcomes: 'no recurrence within 5 years' and 'recurrence within 5 years' were explained. Third, the probabilities of each outcome were given for the 2 scenario descriptions. Both the chances of recurrence and of no recurrence were presented, i.e., a mixed frame was used to avoid the effect of framing (O'Connor, 1989). A period of 5 years was chosen because the main benefits of adjuvant chemotherapy for recurrence emerge during the first 5 years after treatment (EBCTCG, 1998). Recurrence was defined as the first reappearance of breast cancer at any site (local, contralateral or distant), as is customary in the clinical literature (for example EBCTCG, 1998). We used the recurrence rates of patients with a good prognosis (Fisher et al, 1997), so as not to induce anxiety or distress. For the same reasons the prognosis after recurrence was not stated explicitly. It was emphasized to the patients that the probabilities of recurrence mentioned in the treatment preference method probably did not reflect their actual situation and that their individual benefit from adjuvant chemotherapy was unknown to the researchers.

The treatment preference instrument had been tested on 9 healthy volunteers (3 clinical oncologists and 6 co-workers) for clarity of language, accuracy, and whether it provoked too much distress. It was adapted where necessary.

The patients were asked to think back to the time just after their primary treatment and to imagine what they would have done if they had been presented at that time with a choice between 'adjuvant chemotherapy, regular check-ups' and 'no adjuvant chemotherapy, regular check-ups'. Initially, the chance of 'no recurrence within 5 years' without adjuvant chemotherapy was set at 80% and the chance of 'recurrence within 5 years' at 20% (see the Appendix). The benefit of chemotherapy was shown as an increase in the chance of no recurrence from 80% to 90%, i.e., a decrease in recurrence from 20 to 10%, thus an absolute benefit of adjuvant chemotherapy of 10%. The patient was then asked to choose between the 2 options. Next, the chance of no recurrence without adjuvant chemotherapy was held at 80% while the chance of no recurrence with adjuvant chemotherapy was set at 80% (a benefit of 0%) or at 100% (a benefit of 20%), depending on the patients' initial choice. Then, again following the patients' choice, the chances were set at 85% or 95%, and, finally, fine-tuning was carried out to 1%. The lowest chance of no recurrence at which a patient switches from 'no adjuvant chemotherapy, regular check ups' to 'chemotherapy, regular check ups' is the estimate of the minimum benefit to find adjuvant chemotherapy acceptable, our preference score. It was also possible to refuse chemotherapy if the patient believed that for a benefit of 20% (the upper limit in this study) adjuvant chemotherapy was not worthwhile.

### Statistical analysis

Descriptive statistics were used to examine patient characteristics. The baseline characteristics of the patients in the chemotherapy group and in the no-chemotherapy group were compared using the *t*-test for independent samples, the Mann–Whitney U-test for nonparametric data, and the Chi-square test, where appropriate. If significant differences in baseline characteristics were found between the groups, we looked in each group separately to see whether these characteristics were related to preferences. This was done by means of the Spearman-rank correlation coefficient or the Mann–Whitney U-test, where appropriate.

To explore the effects of the hypothesized determinants: positive experience of the treatment and reconciliation with the treatment decision, the preferences of the patients in the chemotherapy group were compared to the responses from the patients in the no-chemotherapy group at all 3 measurement points using Mann–Whitney U-tests. Furthermore, the stability of the preferences was analysed for each group separately by means of the Friedman test for non-parametric data. The stability of preferences at the individual level was analysed by means of the Spearman-rank correlation coefficient.

Within each patient group, it was examined whether the preferences were determined by demographic variables: age (Spearman-rank correlation coefficient), living with others, being a parent, and having children living at home (Mann–Whitney U-test).

### RESULTS

### Patient characteristics

Between June 1996 and November 1998, 71 early-stage breast cancer patients who were eligible for participation in the chemotherapy group were approached. 11 patients (15%) declined participation, mostly because they were unwilling to talk about their recent diagnosis of cancer. 13 patients (18%) could not be interviewed before the start of the treatment for logistic reasons.* 47 patients participated in the first interview. 4 patients (9%) dropped out between the first and second interview: 2 because chemotherapy was prematurely terminated (leucopenia and temporary deterioration in renal function) and 2 because of psychological problems with the interview unrelated to the treatment preference method.** 5 patients (11%) were excluded from the analyses because their preferences were not available at $T_1$, 3 for logistic reasons and 2 because they found the treatment preference method too distressing at that time. This left 38 patients in the chemotherapy group with a complete series of preferences. The preferences of the patients who dropped out between the first and second interview and of the patients whose preferences were not available at $T_1$ were similar to the preferences of the patients in the ultimate chemotherapy group. 38 patients who were not treated with adjuvant chemotherapy were selected in such a way that they matched the patients in the chemotherapy group as closely as possible.

Table 1 shows clinical information of both patient groups and Table 2 shows the demographics.

The groups were compared for matching variables and other clinical and demographic characteristics. Regarding the matching variables, there were no significant differences between the 2 groups. Regarding the other characteristics, the patients in the chemotherapy group more often had nodal involvement (89% versus 37%, $P < 0.01$), had different disease profiles ($P < 0.01$), were generally younger (42 years ± 5.5 versus 55 years ± 9.3, $P < 0.01$), and more frequently had children living at home (76%

*These patients were interviewed after their first course of chemotherapy. They were excluded in the analyses reported here for reasons of clarity.

**This study is part of a larger study into the stability of breast cancer patients' preferences.

© 2001 Cancer Research Campaign

**Table 1** Clinical characteristics

| | Chemotherapy group ($n$ = 38) | No-chemotherapy group ($n$ = 38) | Test for differences |
|---|---|---|---|
| **Clinical variables** | | | |
| Time from surgery to first interview (in days)[a] | | | NS |
| Mean (SD) | 72 (34.7) | 82 (26.6) | |
| Range | 17–153 | 20–134 | |
| Lymph node metastasis[c] | | | $P < 0.01$ |
| Yes | 34 (89%) | 14 (37%) | |
| Prior therapy[b] | | | NS |
| Lumpectomy and radiotherapy | 15 (39%) | 17 (45%) | |
| Modified Radical Mastectomy and radiotherapy | 12 (32%) | 8 (21%) | |
| Modified Radical Mastectomy without radiotherapy | 11 (29%) | 13 (34%) | |
| Adjuvant therapy | | | |
| CMF | 32 (84%) | – | |
| AC | 5 (13%) | – | |
| FEC | 1 (3%) | – | |
| Tamoxifen | not during chemotherapy | 17 (45%) | |
| Disease profile[b] | | | $P < 0.01$ |
| < 50 and lymph node positive | 31 (82%) | – | |
| ≥ 50 and lymph node positive | 3 (8%) | 14 (37%) | |
| < 50 and lymph node negative | 4 (10%) | 11 (29%) | |
| ≥ 50 and lymph node negative | – | 13 (34%) | |
| Hospital[b] | | | NS |
| Leiden University Medical Center | 11 (29%) | 6 (16%) | |
| Diaconessen Hospital | 10 (26%) | 14 (37%) | |
| Rijnland Hospital | 8 (21%) | 15 (40%) | |
| Reinier de Graaf Gasthuis | 7 (18%) | 3 (8%) | |
| Groene Hart Hospital | 2 (5%) | – | |
| Interviewer[b] | | | NS |
| A | 4 (11%) | 4 (11%) | |
| B | 21 (55%) | 23 (61%) | |
| C | 13 (34%) | 11 (29%) | |

[a]*t*-test; [b]Mann–Whitney U-test; [c]Chi-square test.

**Table 2** Patient demographics

| | Chemotherapy group ($n$ = 38) | No-chemotherapy group ($n$ = 38) | Test for differences |
|---|---|---|---|
| **Demographics** | | | |
| Age[a] | | | $P < 0.01$ |
| Mean (SD) | 42 (5.5) | 55 (9.3) | |
| Range | 29–50 | 38–77 | |
| Marital status[b] | | | NS |
| Married/living together | 30 (79%) | 28 (74%) | |
| Divorced | 5 (13%) | 1 (3%) | |
| Widowed | – | 3 (8%) | |
| Single | 3 (8%) | 6 (16%) | |
| Living with others[c] | | | NS |
| Yes | 34 (90%) | 28 (74%) | |
| Being a parent[c] | | | NS |
| Yes | 33 (87%) | 31 (82%) | |
| Having children living at home[c] | | | $P<0.01$ |
| Yes | 29 (76%) | 15 (39%) | |
| Education[b] | | | NS |
| < 10 years | 10 (26%) | 11 (29%) | |
| 10–15 years | 13 (34%) | 16 (42%) | |
| > 15 years | 15 (39%) | 11 (29%) | |
| Occupational status[b] | | | NS |
| Full-time employment | 14 (37%) | 5 (13%) | |
| Part-time employment | 20 (53%) | 17 (45%) | |
| Housewife | 4 (11%) | 16 (42%) | |

[a]*t*-test; [b]Mann–Whitney U-test; [c]Chi-square test.

versus 39%, $P < 0.01$). These differences were inevitable since at the time of the study the treatment protocol prescribed adjuvant chemotherapy for women with nodal involvement under the age of 50, whereas for those above 50 hormonal therapy was the standard treatment. The potential impact of the differences in clinical and demographic characteristics between both patient groups for the preferences will be discussed later.

### Minimum benefit to find chemotherapy acceptable

The proportion of patients accepting adjuvant chemotherapy in exchange for improved disease-free survival varied according to the magnitude of the potential improvement in disease-free survival (see Figures 1A and 1B).

In the chemotherapy group, 39% of the patients responded that they would be willing to accept adjuvant chemotherapy even if it had no clinical benefit at all (0% benefit), averaged over the 3 measurement points. 1 patient responded that she was not willing to accept chemotherapy at $T_1$ and $T_2$, whereas at $T_3$ 2 patients said that they would refuse chemotherapy for any improvement in disease-free survival.

In the no-chemotherapy group on average (over the 3 measurement points) 8% of the patients said that they would accept chemotherapy for a benefit of 0% (see Figure 1B). About one third of the patients in the no-chemotherapy group ($T_1$: 34%, $T_2$: 29%, $T_3$: 37%) responded that they would refuse chemotherapy no matter what benefit.

 *© 2001 Cancer Research Campaign*

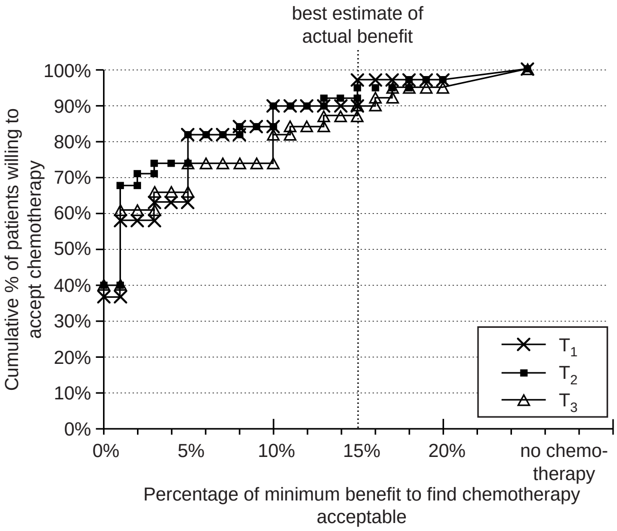

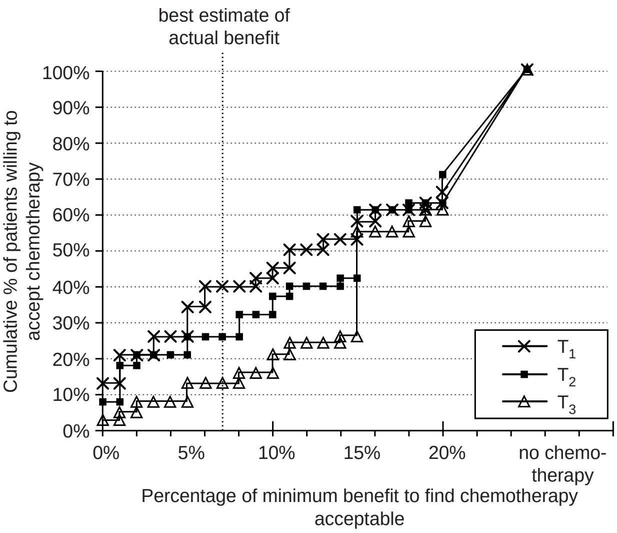

**Figure 1 A** Cumulative proportion of patients in chemotherapy group (*n* = 38) willing to accept adjuvant chemotherapy according to minimum percentage of benefit, measured before ($T_1$), during ($T_2$), and after ($T_3$) chemotherapy. **B** Cumulative proportion of patients in no-chemotherapy group (*n* = 38) willing to accept adjuvant chemotherapy according to minimum percentage of benefit, measured at baseline ($T_1$), three months thereafter ($T_2$), and 6 months thereafter ($T_3$)

### Determinants of treatment preference scores

The median preference scores as well as the interquartile ranges in both patients groups are presented in Table 3. At $T_1$, before the chemotherapy group actually obtained experience with chemotherapy, significant differences were found between the preferences of the patients in the chemotherapy group and those of the patients in the no-chemotherapy group. The patients in the latter group needed more benefit from adjuvant chemotherapy before they would be willing to accept this treatment (median scores: 1% versus 12%, $P < 0.01$). These differences were also found at $T_2$ and $T_3$ (median scores: 1% versus 15%, $P < 0.01$).

In both groups, the preferences remained stable over time. Although the patients in the no-chemotherapy group were somewhat more negative towards chemotherapy at $T_2$ and $T_3$ than at $T_1$, this did not reach statistical significance. In the chemotherapy group, the Spearman-rank correlation coefficient between the preferences obtained at $T_1$ and $T_2$ was 0.66 ($P < 0.01$) and 0.63 ($P < 0.01$) between the preferences obtained at $T_2$ and $T_3$. In the no-chemotherapy group, the Spearman-rank correlation coefficients were 0.57 ($P < 0.01$) and 0.64 ($P < 0.01$), respectively. These results show that the individual preferences are reasonably reliable.

We hypothesized that if having had positive experience with chemotherapy determines preferences, then the preferences of treated patients should become more positive during chemotherapy while the preferences of the patients in the no-chemotherapy group should remain the same over time. If reconciliation with the treatment decision determines the preferences, then we would expect that the preferences of the patients in the chemotherapy group would be more positive towards chemotherapy before the actual start of the treatment than the preferences of the patients in the no-chemotherapy group. The results suggest that reconciliation with the treatment decision is a more important determinant of preferences than positive experience of the treatment.

To support this outcome it has to be ruled out that the differences in preferences between both patient groups are related to the differences in clinical and demographic characteristics that were found between the 2 groups. To examine this, the relationships between the baseline characteristics in question (nodal status, disease profile, age, and having children living at home) and the preferences were analysed, in each group separately. No significant differences in preferences were found between the patients with positive and negative lymph nodes, or between the patients with various disease profiles (based on age and lymph node status). In the no-chemotherapy group, a significant effect ($P < 0.05$) was found for age at $T_2$ (Spearman-rank correlation coefficient: 0.35, $n = 38$), indicating that younger patients would be willing to accept chemotherapy for less benefit than older patients. In the chemotherapy group, no such effect was found. The preferences of patients with children living at home were not significantly different from those without.

Finally, we examined whether the preferences were affected by demographic variables that are known from the literature to be related to the willingness to accept treatment. The variables age and having children living at home have been discussed above, which leaves the variables living with others and being a parent. A Mann–Whitney U-test showed no differences in preferences between patients who lived alone and those who were living with others. Moreover, no significant differences in preferences were found between patients who had children and those without children.

A Mann–Whitney U-test showed that in the no-chemotherapy group the preferences did not differ significantly between patients treated with Tamoxifen and those not treated with Tamoxifen. This supports our assumption that hormonal therapy did not influence decisions for adjuvant chemotherapy.

### DISCUSSION

The purpose of this study was 2-fold. First, to establish the minimum benefits that early-stage breast cancer patients need before they are willing to accept adjuvant chemotherapy. Second, to explore previously described determinants of preferences.

### Minimum benefit to find chemotherapy acceptable

In patients undergoing chemotherapy, the median minimum benefit to find chemotherapy acceptable was 1% at all 3 measurement

© 2001 Cancer Research Campaign

**Table 3** Median scores and interquartile ranges (IQR) of the minimum benefit to find chemotherapy acceptable in early-stage breast cancer patients undergoing adjuvant chemotherapy ($n$ = 38) and not undergoing adjuvant chemotherapy ($n$ = 38). Preferences were elicited before ($T_1$), during ($T_2$), and after ($T_3$) chemotherapy or at similar points in time in the no-chemotherapy group

| | $T_1$ | | $T_2$ | | $T_3$ | | *P* value for differences over time |
|---|---|---|---|---|---|---|---|
| | Median(%) | IQR | Median(%) | IQR | Median(%) | IQR | |
| Chemotherapy group | 1 | 0–5 | 1 | 0–5 | 1 | 0–10 | $P = 0.67$ |
| No-chemotherapy group | 12 | 3–20 | 15 | 5–20 | 15 | 13–20 | $P = 0.10$ |
| *P* value for differences between groups | $P < 0.01$ | | $P < 0.01$ | | $P < 0.01$ | | |

points. On average, 94% of patients indicated a willingness to accept adjuvant chemotherapy for a 15% (or less) improvement in 5-year disease-free survival, which is the mean benefit in 5-year disease-free survival for early-stage breast cancer patients under the age of 50 with positive lymph nodes according to a recent overview of randomized trials (EBCTCG, 1998). There seems to be no doubt that the actual prescription of adjuvant chemotherapy in this patient group was in accordance with the patients' preferences for a hypothetical situation obtained by means of a treatment preference instrument.

In patients not undergoing chemotherapy, the median minimum benefit to find chemotherapy acceptable was 12%, 15% and 15% at $T_1$, $T_2$ and $T_3$, respectively. Because these patients were generally older and were more likely to have negative lymph nodes, their absolute benefit from adjuvant chemotherapy would be less than that of the patients in the chemotherapy group. The best mean estimates of benefits of chemotherapy in 5-year disease-free survival are 6.7%, 9.4% and 6.3% for patients between 50 and 69 years with positive lymph nodes, for patients under the age of 50 with negative lymph nodes, and for patients between 50 and 69 years with negative lymph nodes, respectively (EBCTCG, 1998). Because these subgroups are fairly evenly represented in the no-chemotherapy group (see Table 1), and because no significant differences were found between the preferences of the patients in the various subgroups that were based on disease profiles, the best mean estimate of benefit in the no-chemotherapy group is approximately 7% (the mean benefit of the 3 subgroups).

In this study, on average 26% of the patients in the no-chemotherapy group stated that they would accept adjuvant chemotherapy for a benefit of 7% or less ($T_1$: 39%; $T_2$: 26%; $T_3$: 13%). These results seem to indicate that not prescribing adjuvant chemotherapy in this patient group is according to the preferences of 75% of the patients. However, we cannot be sure that the patients in the no-chemotherapy group would have accepted chemotherapy in a real life situation for less benefit. Would they have accepted chemotherapy if the doctor had proposed it as a real choice? Siminoff and Fetting (1991) found that the strongest predictor for the treatment decisions of patients with a life-threatening illness was the physician's primary treatment recommendation. We do not know whether the patients in our study discussed the option of adjuvant chemotherapy with their specialists. Most likely they were not offered chemotherapy and did not ask for it.

### Determinants of treatment preference scores

Studies have shown that subjects are more willing to accept treatments of which they have personal experience (Yellen et al, 1994; McQuellon et al, 1995; Cullen et al, 1996; Stiggelbout et al, 1996; Lindley et al, 1998; Nieuwkerk et al, 1998). In this study, we examined which was the more important determinant of preferences: (1) positive experience of the treatment or (2) reconciliation with the treatment decision. The first explanation is based on the patients' satisfaction with the treatment that they have experienced. The second implies that patients may have a desire to believe that the prior decision was the correct one (Lindley et al, 1998) and that they will try to justify this decision. This is based on the psychological mechanism referred to as 'cognitive dissonance reduction' (Berkowitz, 1986).

The apparent stability in preferences that was found over time in the chemotherapy group seems to exclude the positive experience of treatment explanation. Moreover, the large differences in preferences between the 2 groups, even before the start of the treatment, seem to support the idea of reconciliation with the treatment decision as a determinant of preferences. In this study, the prior decision refers to the decision to accept chemotherapy by the patients in the chemotherapy group and the decision to accept either no adjuvant treatment or hormonal treatment by the patients in the no-chemotherapy group. After the treatment choice has been made, patients may be unwilling to think about the alternative that was not proposed, but might have been preferable (Cullen et al, 1996), or upon the alternative that was turned down. This may result in more positive preferences for chemotherapy in the chemotherapy group than in the no-chemotherapy group.

With regard to other potential determinants of preferences, our results did not show influences on the patients' preferences of: nodal status, disease profile, treatment with Tamoxifen, living with others, being a parent, and finally, having children living at home. The observation that the patients in the chemotherapy group were significantly younger than those in the no-chemotherapy group might have had an impact on their preferences. However, a statistically significant relationship between age and preferences was found only at $T_2$ in the no-chemotherapy group. This relationship was moderate ($r = 0.35$, $P < 0.05$). Previous studies that observed relationships between age and treatment preferences have also found only moderate relationships. Brundage et al (1997) observed a non-significant tendency that a higher proportion of patients younger than 60 years would accept combined chemoradiotherapy as compared with older patients. In the study by McQuellon et al (1995), younger subjects were more likely to accept treatment, but this effect was modest and observed only for a prolongation of survival of 6 months and not in the case of 5 years, 18 months, 1 year, 1 month and 1 week. Yellen et al (1994) concluded that older patients differ in terms of willingness to trade survival for current quality of life only when treatment is presumed and not in terms of acceptance of chemotherapy. The results of these studies, as well as the absence of an association at

*© 2001 Cancer Research Campaign*

$T_1$ and $T_3$, may support our assumption that the differences in preferences between the 2 groups may only to a small extent be explained by differences in age.

There may, however, be other aspects that determine preferences than we have explored in this study. According to Yellen and Cella (1995), the most important predictor of whether a patient will accept a potentially aggressive treatment may be the way in which the treatment is described by the oncologist, and how strongly it is recommended. This may have played a role in our study results because the preferences were elicited after the actual decision for treatment was made. Most probably, the specialists' recommendations for adjuvant chemotherapy were stronger in the chemotherapy group than in the no-chemotherapy group.

### Accepting adjuvant chemotherapy for no clinical benefit

The most remarkable observation we made is that almost 40% of the patients undergoing chemotherapy and 8% of the patients not eligible for chemotherapy responded that they would accept chemotherapy if it had no clinical benefit at all. Similar findings were observed in other studies. Yellen and Cella (1995) observed that 45% of cancer patients stated that they would accept mildly toxic adjuvant therapy for 1% increase in likelihood of cure. In the study by Ravdin et al (1998), the median acceptable reduction of risk of recurrence at 5 years of follow-up was 0.5% to 1% in early-stage breast cancer patients who previously had received adjuvant chemotherapy. Finally, Palda et al (1997) observed that 46% of breast cancer patients would not give up post-operative radiotherapy, even in the face of no stated benefit.

In the present paper, the benefit of adjuvant chemotherapy is described in terms of an improvement in 5-year disease-free survival, thus a clinically defined benefit. However, adjuvant chemotherapy may have other benefits for patients. For example, Levine et al (1988) observed, through unstructured interviews, that chemotherapy can provide women with a sense of control over their lives and with a feeling that they are doing something active to deal with their disease. Chemotherapy may help to handle the feeling of helplessness that accompanies the diagnosis of breast cancer. Palda et al (1997) observed that 57% of patients who were willing to accept post-operative radiotherapy for zero benefit, had a positive, persistent belief in the treatment benefits: they 'felt better' or were 'convinced/know/believe/certain that it will help'. Other patients used more negative terms, such as 'worry', 'fear', 'regret' and 'doubt'. These kinds of motivations will also have contributed to the acceptance of chemotherapy for small or no clinical benefit in this study. Our patients remarked spontaneously that this was the case; e.g., 'At least, I have done everything I could'. In addition, anticipated regret (Bell, 1982) seems to have played a role; 'In the future, I do not want to regret having refused chemotherapy'.

If we adhere to patient autonomy and shared decision-making, are we willing to give burdening treatments to patients for cognitive and affective reasons? Since more and more attention is focused on shared decision-making and patient autonomy, further research into the (ir)rationality of patients' preferences will be necessary. Patients can make choices that are based on criteria or points of views that may seem irrational to the outsider. If a patient's goal is to live at all costs and if she believes that chemotherapy may help her, no matter what the doctors say, the choice to accept chemotherapy may be highly rational. However, if we define a rational decision as one that complies with the principles of expected utility, it is clear that accepting a potential aggressive treatment without expected benefit is not a rational choice. To what extent should a health care system, or society, tolerate such 'irrational' choices by well-informed and autonomous patients? Subsequently, if there are limits to the 'irrationality' that society tolerates, and is willing to pay for, who is going to set these limits, and how? These questions need to be solved before the 'irrational' choices we found can be dealt with.

### Limitations and future directions

The generalization of our study results is limited by the differences between both patients groups with respect to clinical and demograpical characteristics. Another limitation is that the minimum benefit to find adjuvant chemotherapy acceptable is assessed in a hypothetical situation and may not reflect the minimum benefits the patients would need in a real-life situation. As a consequence of the process of reconciliation with the treatment decision the preferences presented here may be too positive in the patients undergoing chemotherapy and too negative in the patients not scheduled for chemotherapy. Studies into treatment preferences have been carried out in patients who have already undergone the particular treatment (Nieuwkerk et al, 1998; Ravdin et al, 1998; Silvestri et al, 1998), in mixed patient groups with some patients having experience and others not (O'Connor, 1989; Kiebert et al, 1993; Yellen et al, 1994; McQuellon et al, 1995; Yellen and Cella, 1995; Cullen et al, 1996; Elit et al, 1996; Brundage et al, 1997; Lindley et al, 1998), in patients who were about to undergo the specific treatment (Slevin et al, 1990; Palda et al, 1997), or in respondents who did not receive the treatment under concern (McNeil et al, 1982; Llewellyn-Thomas et al, 1996). As a result, all studies mentioned above have been carried out in a hypothetical situation and may have been influenced by the effect of 'reconciliation with the treatment decision'. The finding in our study that 'reconciliation with the treatment decision' may have such a large impact on the preferences for treatment has important consequences for the field of oncology in that the results of previously published preference studies must be interpreted with caution. As far as we know only 2 pilot studies have used a treatment preference instrument in an actual decision-making situation to assist women with breast cancer to decide whether or not to receive post-operative radiotherapy (Whelan et al, 1995) or adjuvant chemotherapy (Levine et al, 1992). We recommend that future studies into patients' preferences be performed in patients before the actual treatment decision has been made. By repeating the measurements after the treatment decision the potential impact of 'reconciliation with the treatment decision' can be determined in other patient groups and with other treatments.

In conclusion, in decision-making on adjuvant chemotherapy for breast cancer both patients and doctors should be aware that patients' preferences may not only be based on clinical factors but also be influenced by cognitive and affective factors. Moreover, these determinants may have such a strong impact that even treatment without obvious clinical benefit, and with possible side effects, may be considered worthwhile by patients.

### ACKNOWLEDGEMENTS

The authors thank the oncologists of the participating hospitals for their support in approaching their patients and the patients for their contribution to this study. Supported by a grant from the Dutch Cancer Society (Grant 95-734).

© 2001 Cancer Research Campaign 

### REFERENCES

Bell DE (1982) Regret in decision making under uncertainty. *Oper Res* **30**: 961–981

Berkowitz L (1986) *A survey of social psychology* (3rd ed), pp. 64. Holt, Rinehart and Winston: New York

Brundage MD, Davidson JR and Mackillop WJ (1997) Trading treatment toxicity for survival in locally advanced non-small cell lung cancer. *J Clin Oncol* **15**: 330–340

Cullen MH, Billingham LJ, Cook J and Woodroffe CM (1996) Management preferences in stage I non-seminomatous germ cell tumours of the testis: an investigation among patients, controls and oncologists. *Br J Cancer* **74**: 1487–1491

Dodwell DJ (1998) Adjuvant cytotoxic chemotherapy for early breast cancer: doubts and decisions. *Lancet* **351**: 1506–1507

Early Breast Cancer Trialists' Collaborative Group (1998) Polychemotherapy for early breast cancer: an overview of the randomised trials. *Lancet* **352**: 930–942

Elit LM, Levine MN, Gafni A, Whelan TJ, Doig G, Streiner DL and Rosen B (1996) Patients' preferences for therapy in advanced epithelial ovarian cancer: development, testing, and application of a bedside decision instrument. *Gynecologic Oncology* **62**: 329–335

Fisher B, Dignam J, Wolmark N, DeCillis A, Emir B, Wickerham DL, et al (1997) Tamoxifen and chemotherapy for lymph node-negative, estrogen receptor-positive breast cancer. *J Natl Cancer Inst* **89**: 1673–1682

Jansen SJT, Stiggelbout AM, Nooij MA and Kievit J (2000) The effect of individually assessed preference weights on the relationship between holistic utilities and nonpreference-based assessment. *Quality Life Res* **9**: 541–557

Kiebert GM, Stiggelbout AM, Leer JWH, Kievit J and de Haes JCJM (1993) Test-retest reliabilities of two treatment-preference instruments in measuring utilities. *Med Decis Making* **13**: 133–140

Levine MN, Guyatt, GH, Gent M, De Pauw S, Goodyear MD, Hryniuk WM, et al (1988) Quality of life in stage II breast cancer: an instrument for clinical trials. *J Clin Oncol* **6**: 1798–1810

Levine MN, Gafni A, Markham B and MacFarlane D (1992) A bedside decision instrument to elicit a patient's preference concerning adjuvant chemotherapy for breast cancer. *Ann Intern Med* **117**: 53–58

Lindley C, Vasa S, Sawyer WT and Winer EP (1998) Quality of life and preferences for treatment following systemic adjuvant therapy for early-stage breast cancer. *J Clin Oncol* **16**: 1380–1387

Llewellyn-Thomas HA (1997) Investigating patients' preferences for different treatment options. *Can J Nurs Res* **29**: 45–64

Llewellyn-Thomas HA, Williams JI, Levy L and Naylor CD (1996) Using a trade-off technique to assess patients' treatment preferences for benign prostatic hyperplasia. *Med Decis Making* **16**: 262–272

McNeil BJ, Pauker SG, Sox HC Jr and Tversky A (1982) On the elicitation of preferences for alternative therapies. *N Engl J Med* **306**: 1259–1262

McQuellon RP, Muss HB, Hoffman SL, Russell G, Craven B and Yellen SB (1995) Patient preferences for treatment of metastatic breast cancer: a study of women with early-stage breast cancer. *J Clin Oncol* **13**: 858–868

Nieuwkerk PT, Hajenius PJ, Van der Veen F, Ankum WM, Wijker W and Bossuyt PM (1998) Systemic methotrexate therapy versus laparoscopic salpingostomy in tubal pregnancy. Part II. Patient preferences for systemic methotrexate. *Fertility and Sterility* **70**: 518–522

O'Connor AM (1989) Effects of framing and level of probability on patients' preferences for cancer chemotherapy. *J Clin Epidemiol* **42**: 119–126

Palda VA, Llewellyn-Thomas HA, Mackenzie RG, Pritchard KI and Naylor CD (1997) Breast cancer patients' attitudes about rationing postlumpectomy radiation therapy: applicability of trade-off methods to policy-making. *J Clin Oncol* **15**: 3192–3200

Pritchard K (1997) Breast cancer: the real challenge. *Lancet* **349**: sI24–sI26

Rajagopal S, Goodman PJ and Tannock IF (1994) Adjuvant chemotherapy for breast cancer: discordance between physicians' perception of benefit and the results of clinical trials. *J Clin Oncol* **12**: 1296–1304

Ravdin PM, Siminoff IA and Harvey JA (1998) Survey of breast cancer patients concerning their knowledge and expectations of adjuvant therapy. *J Clin Oncol* **16**: 515–521

Sainsbury R, Rider L, Smith A and MacAdam A, on behalf of the Yorkshire Breast Cancer Group (1995) Does it matter where you live? Treatment variation for breast cancer in Yorkshire. *Br J Cancer* **71**: 1275–1278

Silvestri G, Pritchard R and Welch HG (1998) Preferences for chemotherapy in patients with advanced non-small cell lung cancer: descriptive study based on scripted interviews. *BMJ* **317**: 771–775

Siminoff LA and Fetting JH (1991) Factors affecting treatment decisions for a life-threatening illness: the case of medial treatment of breast cancer. *Soc Sci Med* **32**: 813–818

Slevin ML, Stubbs L, Plant HJ, Wilson P, Gregory WM, Armes PJ, et al (1990) Attitudes to chemotherapy: comparing views of patients with cancer with those of doctors, nurses, and general public. *BMJ* **300**: 1458–1460

Stiggelbout AM, Kiebert GM, de Haes JC, Keizer HJ, Stoter G, de Wit RS, et al (1996) Surveillance versus adjuvant chemotherapy in stage I non-seminomatous testicular cancer: a decision analysis. *Eur J Cancer* **32A**: 2267–2274

Stiggelbout AM, de Haes JC, and van de Velde CJ (2000) Adjuvant chemotherapy in node negative breast cancer: patterns of use and oncologists' preferences. *Ann Oncol* **11**: 631–633

Whelan TJ, Levine MN, Gafni A, Lukka H, Mohide EA, Patel M and Streiner DL (1995) Breast irradiation postlumpectomy: development and evaluation of a decision instrument. *J Clin Oncol* **13**: 847–853

Yellen SB, Cella DF and Leslie WT (1994) Age and clinical decision making in oncology patients. *J Natl Cancer Inst* **86**: 1766–1770

Yellen SB and Cella DF (1995) Someone to live for: social well-being, parenthood status, and decision-making in oncology. *J Clin Oncol* **13**: 1255–1264

*© 2001 Cancer Research Campaign*

### APPENDIX

Description of treatment preference method. Chances of recurrence and of no-recurrence for 'no chemotherapy, regular check-ups' are held constant while chances of recurrence and of no-recurrence for 'chemotherapy, regular check-ups' are varied according to respondent's preferences. The point where the respondent switches from 'no chemotherapy' to 'chemotherapy' indicates the minimum benefit to find chemotherapy acceptable.

| Treatment option | Chance of outcome | Outcome |
|---|---|---|
| **No chemotherapy, regular check-ups**<br>Each check-up includes a physical examination. A mammogram is made once a year.<br><br>The side effects and especially the psychological and social consequences as mentioned with chemotherapy may also occur in this option as a result of previous surgery, radiotherapy or the diagnosis of cancer. | 80% → | **No recurrence within 5 years**<br>All tests and examinations show that there is no recurrence of the cancer. One may sometimes worry that the tumour may return. |
| | 20% → | **Recurrence within 5 years**<br>The tumour may recur in the breast or at a different site in the body. Various treatments may follow like radiotherapy, chemotherapy or hormonal therapy. The prognosis is uncertain. |
| **Chemotherapy, regular check-ups**<br>During 6 months 1 or 2 hospital visits per 3 weeks for chemotherapy treatment intravenously.<br>During and after chemotherapy regular check-ups.<br>The following side effects and psychological and social consequences may occur:<br>*Physical*: Nausea, fatigue, hair loss, difficulty in carrying out strenuous activities, frequent need to rest<br>*Psychological*: Dissatisfaction with one's body<br>*Social*: Limitations to work or other daily activities, restrictions on social | 90% → | **No recurrence within 5 years**<br>All tests and examinations show that there is no recurrence of the cancer. One may sometimes worry that the tumour may return. |
| | 10% → | **Recurrence within 5 years**<br>The tumour may recur in the breast or at a different site in the body. Various treatments may follow like radiotherapy, chemotherapy or hormonal therapy. The prognosis is uncertain. |

© 2001 Cancer Research Campaign 
